# Bone marrow function in cervical cancer patients after concurrent chemoradiotherapy using ^99m^Tc-SC SPECT/CT: A cross-sectional retrospective study

**DOI:** 10.1097/MD.0000000000047766

**Published:** 2026-02-20

**Authors:** Chunmeng Chen, ShanBing Wang, Jiapei Liu, Jie Liao, Jianqiang Wang, Qi Yang

**Affiliations:** a Department of Nuclear Medicine, the Second People’s Hospital, Yibin, Sichuan, China; b Department of Oncology, the Second People’s Hospital of Yibin City, Yibin, Sichuan, China; c Department of Laboratory Medicine, the Second People’s Hospital of Yibin City, Yibin, Sichuan, China.

**Keywords:** ^99m^Tc-sulfur colloid, bone marrow function, cervical cancer, radiotherapy and chemotherapy, SPECT/CT

## Abstract

^99m^Tc-sulfur colloid (^99m^Tc-SC) single-photon computed emission tomography/computed tomography (SPECT/CT) bone marrow (BM) scintigraphy is a key diagnostic tool for distinguishing active red BM from inactive yellow BM. Although previous research has documented that it could reduce the volume of ABM irradiated at higher doses, the role of SPECT/CT parameters in assessing BM function in different pelvic regions and in predicting hematologic toxicity (HT) remains underexplored. This study aimed to investigate the value of ^99m^Tc-SC SPECT/CT imaging for assessing BM function and its predictive role for HT in patients with cervical cancer undergoing concurrent chemoradiotherapy (CRT). In this retrospective study, 40 patients with stage IB2-IVA cervical cancer underwent ^99m^Tc-SC SPECT/CT before and within 2 weeks after CRT. Patients were stratified into BM suppression (BMS) and non-BMS groups based on grade ≥ 3 HT. Semi-quantitative uptake ratios (R, liver-normalized) and their changes (ΔR) across 5 pelvic sites (L4, L5, sacrum, ilium, pubis) were compared. Analyses incorporated False Discovery Rate correction for multiple comparisons, non-parametric sensitivity tests, and effect size reporting. Predictive performance for HT was assessed using receiver operating characteristic curves and internally validated via leave-one-out cross-validation. CRT induces a spatially heterogeneous suppression of BM function, predominantly affecting the lumbosacral region. Elevated semi-quantitative uptake on pretreatment ^99m^Tc-SC SPECT/CT, particularly in the lumbosacral region, exhibits potential as a biomarker for predicting severe HT. These findings underscore the promise of functional imaging for personalized risk stratification, pending validation in larger, prospective, multi-institutional cohorts. Post-CRT reduction in BM uptake (ΔR) was significantly more substantial within the lumbosacral spine (L4, L5, Sacrum) compared to the pelvic bones (Ilium, Pubis), with a moderate overall effect (partial η^2^ = 0.156). Patients who developed grade ≥ 3 HT (BMS group) exhibited significantly higher pretreatment *R* values and greater ΔR declines in the lumbosacral spine compared to the non-BMS group, with large effect sizes (e.g., Cohen d up to −1.05). Pretreatment *R* values at L4 (area under the curve [AUC] = 0.769), L5 (AUC = 0.767), and sacrum (AUC = 0.793) were significant predictors of grade ≥ 3 HT, a finding affirmed by leave-one-out cross-validation (cross-validated AUCs: 0.724, 0.737, and 0.752, respectively).

## 1. Introduction

Cervical cancer is a major contributor to female cancer-related deaths.^[1]^ Typically, locally advanced cervical cancer is subjected to standard treatment for tumor control using a multimodal approach, consisting of external-beam radiation therapy with concomitant chemotherapy, followed by brachytherapy.^[2]^ However, a major shortcoming of this therapy is treatment-related side effects in the surrounding healthy tissues. Prospective data have estimated an incidence ranging between 20% and 25% for grade ≥ 3 hematologic toxicity (HT) in platinum-based pelvic chemoradiation.^[3]^ During the treatment of cervical cancer patients, the radiotherapy target volume covers areas containing > 50% of active bone marrow (ABM, primarily regions such as the lumbar spine and pelvis). Therefore, it plays a pivotal role in replenishing blood cells, and the bone marrow (BM) sustains varying degrees of damage during chemoradiotherapy (CRT).^[4,5]^ Moreover, extended-field para-aortic radiation with increased exposure of the pelvic and lower spine bones may result in even broader exposure of the overall BM, leading to an elevated incidence of HT.^[3]^ Consequently, it may trigger a series of hematological adverse reactions characterized by neutropenia, ultimately treatment interruptions, increased demand for antibiotics and growth factors, and occasionally even severe infections and related deaths.^[6]^

With the advancement of imaging techniques, ^99m^Tc-sulfur colloid (^99m^Tc-SC) single-photon computed emission tomography/ computed tomography (SPECT/CT) BM scintigraphy has been accepted as a powerful tool to assist clinicians in the optimization of BM protection plans for patients with tumors. Given that ^99m^Tc-SC is sequestered by macrophages associated with red marrow, this type of imaging can effectively distinguish active red BM from inactive yellow BM. Imaging using SPECT/CT can provide a 3-dimensional map of the distribution of ABM regions.^[7]^ According to existing publications, SPECT/CT-defined subregions could reduce the volume of ABM irradiated at higher doses, which could facilitate ABM sparing and improve the predictive value of acute HT classified as grade ≥ 3.^[7–10]^ For instance, Wang et al showed that SPECT/CT-defined ABM-sparing volumetric-modulated arc therapy (VMAT) can reduce the incidence of acute HT in patients with locally advanced cervical cancer.^[9]^ However, there is currently no relevant research investigating the role of SPECT/CT parameters in assessing BM function in different pelvic regions and in predicting HT in patients with cervical cancer during CRT. Moreover, the role and relevant mechanisms of SPECT/CT parameters in predicting HT remain unclear.

Therefore, while ^99m^Tc-SC SPECT/CT has been integrated into planning for anatomical BM sparing, its potential to serve as a functional biomarker, quantifying baseline marrow activity to predict individual susceptibility to CRT-induced HT, is not well established. A critical gap exists in understanding whether the pre-therapeutic functional intensity of active marrow, which may reflect its proliferative state, correlates with subsequent hematologic risk. Consequently, this study aimed to assess the regional heterogeneity of BM functional suppression after CRT using ^99m^Tc-SC SPECT/CT and to evaluate the hypothesis that semi-quantitative baseline uptake parameters are associated with the likelihood of developing severe (grade ≥ 3) HT.

## 2. Methods

### 2.1. Eligibility criteria

A retrospective observational study was conducted on patients with histologically confirmed cervical cancer, with inclusion dates ranging from January 2017 to June 2023. All the patients signed an informed consent form. The inclusion criteria were as follows: age 18 to 70 years; pathologically confirmed locally advanced cervical cancer (FIGO 2008 stages IB2, IIA2, IIB, III, and IVA); life expectancy > 12 months; and no prior radiotherapy or chemotherapy. Patients with other pelvic malignancies, hematologic malignancies, skeletal tumors, primary liver diseases (e.g., cirrhosis and liver cancer), or active infectious diseases were also excluded. Ethical approval by the Ethics Committee of this hospital was obtained before the initiation of this study, and all patients provided informed consent before the examination.

### 2.2. Treatment planning

All patients underwent concurrent CRT, with no cases of HT or infection-related treatment interruption. Chemotherapy consisting of weekly cisplatin (30–40 mg/m^2^) alone was administered during pelvic external beam radiation therapy (EBRT). The clinical target volume (CTV) covered the uterus, cervix, gross tumor parametria, adequate vaginal margin from the gross disease (at least 3 cm), and regional nodes (common, internal, and external iliac nodes, obturator nodes, and bilateral groin nodes in patients with lower 1/3 vaginal involvement). The planning target volume (PTV) was generated uniformly through extension of the CTV by 6 mm in each direction. The VMAT plans were developed using the Monaco treatment planning system (Elekta AB, Stockholm, Sweden). The patients received 45 Gy in 25 fractions to the PTV. High-dose-rate (HDR) intracavitary brachytherapy was delivered twice weekly after EBRT. The typical A prescribed dose was 5 fractions of 6 Gy per fraction at an HDR. In addition, boosts of an additional 10 to 20 Gy were administered to patients with pelvic lymph nodes 2 weeks after the end of CRT. All VMAT plans were normalized to cover approximately 98% of the PTV with a prescription dose (45Gy).

### 2.3. ^99m^Tc-SC SPECT/CT

With no specific preparation required, all patients underwent ^99m^Tc-SC SPECT/CT BM scintigraphy before and within 2 weeks after CRT. One hour before imaging, each patient was intravenously injected with 99mTc sulfur colloid (7.4 MBq/kg) in the supine position for subsequent SPECT/CT (GE Infinia Hawkeye 4) imaging. Each patient underwent SPECT/CT from the liver to the bilateral upper femurs (slice thickness, 5 mm), followed by whole-body SPECT/CT imaging (256 × 256 matrix). The automatic rigid registration of all SPECT/CT fused images was based on the bone structure, with manual checking of the coincidence degree of the 2 groups. Notably, the deviation can be manually moved or rotated during the registration.

### 2.4. Image analysis

All examinations were read in consensus by 2 board-certified nuclear medicine physicians with SPECT/CT experience. A visual assessment method was employed for qualitative analysis: grade 0, No BM activity detected in the radiotherapy target area, with radiopharmaceutical distribution resembling the surrounding soft tissues; grade 1, Faint BM activity discernible, slightly elevated above the background of adjacent soft tissues; grade 2, Light BM activity with indistinct contours; and grade 3, Clear BM activity with well-defined contours. Grades 0 to 1 were considered BM suppression (BMS), while Grades 2 to 3 were non-BMS (NBMS). Furthermore, the region of interest (ROI) method was used to delineate and obtain the maximum counts (Cmax) of radioactive uptake in the liver, L4 vertebra, L5 vertebra, sacrum, iliac bones, and pubis. Simultaneously, this study calculated the semi-quantitative values of R (Cmax/liver maximum count) and the difference (ΔR) before treatment (BT) and after treatment (AT).

### 2.5. HT assessment

Complete blood counts were performed weekly for all patients, 1 week before treatment and within 2 weeks of CRT. HT was assessed using the Common Terminology Criteria.^[11]^ HT grade ≥ 3 (HT 3+) was defined as any grade ≥ 3 toxicity for white blood cells, absolute neutrophil count, platelets, and hemoglobin. Patients with an HT grade ≥ 3 (HT 3+) were classified as having BMS. The primary study endpoint was complete blood count results obtained within 2 weeks of CRT.

### 2.6. Statistical analysis

Continuous data were presented as mean ± standard deviation. The difference in treatment response (ΔR) among the 5 anatomical sites (L4, L5, sacrum, ilium, pubis) was analyzed using 1-way analysis of variance (ANOVA). Upon obtaining a significant result, post hoc pairwise comparisons were conducted using Tukey Honestly Significant Difference test. The effect size for ANOVA was reported as partial eta-squared (η^2^p). The non-parametric Kruskal–Wallis test, followed by Dunn post hoc test with Bonferroni correction, was used to confirm the findings. To control for multiple comparisons in post hoc testing, the false discovery rate was also applied using the Benjamini-Hochberg method. Group comparisons between BMS and NBMS patients for various parameters were performed using independent samples *t*-tests. To ensure robustness, Mann–Whitney *U* tests were conducted in parallel for all comparisons. For statistically significant results (t-test *P* < .05), the effect size was calculated using Cohen *d*. Receiver operating characteristic curve analysis was used to evaluate the predictive performance of pretreatment parameters for grade ≥ 3 HT. The area under the curve (AUC) was reported with its 95% confidence interval (CI). To assess model stability and mitigate overfitting, leave-one-out cross-validation (LOOCV) was performed, and the cross-validated AUC (cvAUC) with its 95% CI was calculated. All statistical analyses were performed using SPSS (version 22.0; IBM, Armonk), with a 2-sided *P* value < .05 considered significant.

## 3. Results

This cross-sectional study analyzed 40 patients with cervical cancer (mean age: 53.1 ± 6.8 years) from January 2017 to June 2023. According to the FIGO staging system, 2 patients had stage IA2, 3 had stage IB2, 5 had stage IIA1, 5 had stage IIA2, 20 had stage IIB, 1 had stage IIIA, 3 had stage IIIB, and 1 had stage IV. The average radiation dose received by the pelvic region was 78.36 ± 19.44Gy.

**Figure 1.**
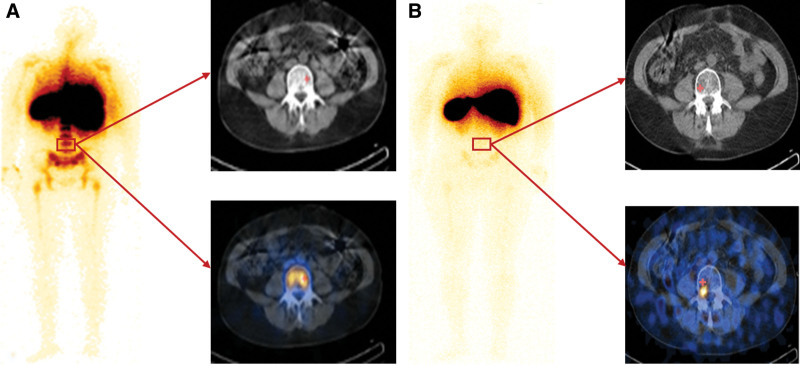
A 46-year-old female patient with moderately differentiated squamous cell carcinoma of the cervix following surgery (FIGO stage IB2). BM scintigraphy showed uniform high uptake in the lumbar region before CRT treatment (A, a qualitative grading of grade 3) and little uptake after treatment (B, a qualitative grading of grade 0). BM = bone marrow, CRT = chemoradiotherapy.

Among the 40 patients, only 4 exhibited grade 2 HT before treatment, 17 showed grade 1 HT, and 19 had no HT. BM imaging revealed qualitative grading of grade 3 in 36 patients and grade 2 in 4 patients. None of the patients had a BMS before treatment. Both the HT and BM grades decreased after treatment. Seventeen patients developed grade 2 HT, while 23 did not. Twenty-one patients had a BM grade of 0 to 1, and 19 patients had a grade 2. The pre- and post-treatment HT and BM qualitative grades are summarized in Table 1. Figure 1 shows representative planar and fused SPECT/CT images demonstrating the changes in BM function before and after CRT treatment. There were no statistically significant differences in the radiation therapy doses administered, FIGO stage, or age between the BMS and NBMS groups (all *P > *.05).

**Table 1:** Number of participants with qualitative grading of HT and BM before and after treatment.

Characteristic	Grade	Number (BT)	Number (AT)
HT	0	19	0
	1	17	6
	2	4	17
	3	0	17
	4	0	0
Qualitative grading of BM	3	36	0
	2	4	19
	1	0	18
	0	0	3

AT = after treatment, BM = bone marrow, BT = before treatment, HT = hematologic toxicity.

To compare the treatment responses (Δ*R* values) among the 5 anatomical sites (Table 2), a 1-way ANOVA was performed. The analysis revealed a statistically significant overall difference (F(4, 195) = 9.010, *P* < .001), with a moderate effect size (partial η^2^ = 0.156). post hoc pairwise comparisons using Tukey Honestly Significant Difference test indicated that the ΔR values of the L4, L5, and sacral vertebrae were significantly higher than those of the iliac and pubic bones. Specifically, the Δ*R* values for L4 and L5 were significantly greater than those for the ilium (both *P* < .01) and pubic bone (both *P* < .001). No statistically significant differences were observed in Δ*R* values among the L4, L5, and sacral sites (all *P* > .05), or between the ilium and pubic bone (*P* > .05). A sensitivity analysis using the non-parametric Kruskal–Wallis test confirmed the significant overall difference among sites (χ^2^(4) = 35.14, *P* < .001). post hoc Dunn tests with Bonferroni correction revealed the same pattern of significant pairwise differences for L4/L5 vs ilium/pubic bone (all adjusted *P* < .05). The results of all pairwise comparisons, including adjusted *P*-values after applying the Benjamini–Hochberg false discovery rate correction, are detailed in Table S1, Supplemental Digital Content, https://links.lww.com/MD/R412.

**Table 2:** Comparison of semi-quantitative *R* values before and after treatment in different regions.

Site	*R* (BT) (Mean ± SD)	*R* (AT) (Mean ± SD)	Δ*R* (Mean ± SD)	*T* value	*P* value
L4	0.087 ± 0.28	0.041 ± 0.014	0.046 ± 0.028	10.499	<.001
L5	0.079 ± 0.034	0.032 ± 0.014	0.048 ± 0.034	8.800	<.001
Sacrum	0.074 ± 0.031	0.034 ± 0.014	0.040 ± 0.027	9.252	<.001
Ilium	0.067 ± 0.029	0.044 ± 0.016	0.023 ± 0.026	5.651	<.001
Pubis	0.044 ± 0.016	0.025 ± 0.012	0.019 ± 0.020	6.277	<.001

AT = after treatment, BT = before treatment.

The patients were further divided into BMS (n = 17) and NBMS (n = 23) groups based on the development of grade ≥ 3 HT (Table 3). Comparative analysis revealed a distinct pattern: the NBMS group exhibited significantly lower baseline (pre-CRT) BM uptake (*R* values) at the axial sites (L4, L5, and sacrum) compared to the BMS group (e.g., L4 R(BT): 0.075 ± 0.023 vs 0.102 ± 0.028, *P* = .004; Table 3). Furthermore, the post-treatment reduction in marrow activity (Δ*R*) was also more pronounced in the BMS group at these sites (all *P* < .05). A similar trend was observed for the iliac and pubic bones, where baseline *R* values were lower in the NBMS group, although the differences in Δ*R* did not reach statistical significance (*P* = .10 and .05, respectively)

**Table 3:** Differences in parameters among BMS and NBMS groups in various regions

Site	NBMS (mean ± SD)	BMS (mean ± SD)	*P* value	Cohen *d*	Mann–Whitney *P*
L4 R(BT)	0.075 ± 0.023	0.102 ± 0.028	.004	−1.021	.003
L4 R(AT)	0.039 ± 0.017	0.043 ± 0.006	.35	−0.287	.07
L4 ΔR	0.036 ± 0.023	0.059 ± 0.029	.012	−0.868	.011
L5 R(BT)	0.065 ± 0.024	0.098 ± 0.037	.004	−1.046	.004
L5 R(AT)	0.032 ± 0.017	0.032 ± 0.010	.89	−0.043	.35
L5 ΔR	0.034 ± 0.023	0.066 ± 0.038	.005	−1.025	.004
Sacral R(BT)	0.061 ± 0.023	0.091 ± 0.033	.004	−1.040	.001
Sacral R(AT)	0.032 ± 0.016	0.037 ± 0.010	.27	−0.350	.045
Sacral ΔR	0.029 ± 0.019	0.054 ± 0.031	.007	−0.980	.004
Iliac R(BT)	0.056 ± 0.020	0.082 ± 0.033	.008	−0.961	.003
Iliac R(AT)	0.039 ± 0.017	0.051 ± 0.011	.01	−0.805	.09
Iliac ΔR	0.017 ± 0.019	0.032 ± 0.032	.10	−0.560	.005
Pubic R(BT)	0.039 ± 0.010	0.052 ± 0.019	.016	−0.870	.005
Pubic R(AT)	0.025 ± 0.012	0.024 ± 0.012	.96	0.017	.93
Pubic ΔR	0.013 ± 0.011	0.028 ± 0.027	.05	−0.691	.11

AT = after treatment, BMS = bone marrow suppression, BT = before treatment, NBMS = non-bone marrow suppression.

Comparative analysis between the BMS and NBMS groups revealed a notable pattern in BM activity parameters (Table 3). All group comparisons were performed using independent samples t-tests. To ensure robustness against potential violations of normality assumptions and to provide non-parametric support, Mann–Whitney *U* tests were conducted in parallel. Effect sizes (Cohen *d*) were calculated for all comparisons where the *t*-test was significant (*P* < .05). The non-parametric analyses yielded consistent results with the parametric t-tests for all significant comparisons. The comprehensive results are presented in Table 3.

The qualitative grading of BM planar imaging was negatively correlated with the HT grading (*r* = −0.715, *P* < .001). The pretreatment *R* values were negatively correlated with post-treatment complete blood count indices (*r* = −0.524, −0.474, −0.506, −0.478, and − 0.0343; *P *< .001). Among these, the strongest correlation was observed for the semi-quantitative *R* value for L4.

**Figure 2.**
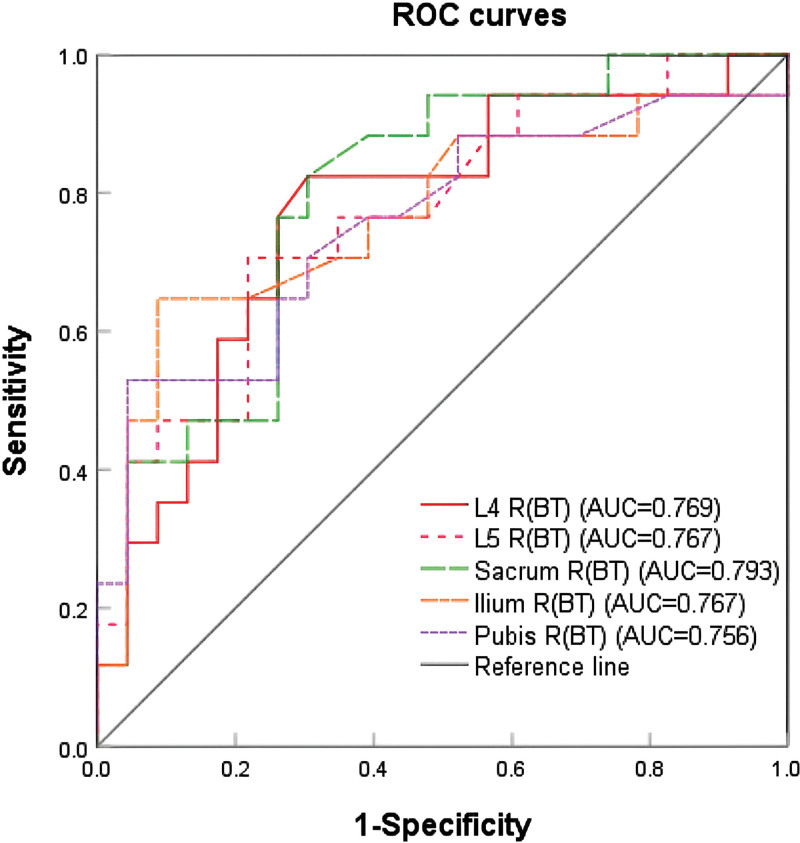
The ROC curves of L4 R, L5 R, sacral R, iliac R, and pubic R before treatment. AUC = area under the curve, ROC = receiver operating characteristic.

Table 4 summarizes the receiver operating characteristic curve analysis for predicting grade ≥ 3 HT. Parameters such as L4 R(AT), L5 R(AT), and pelvic bone ΔR values failed to demonstrate significant predictive value. In contrast, pretreatment R values at all 5 sites showed significant predictive ability (Fig. 2) : L4 R (cutoff ≥ 0.089, AUC = 0.769, *P* = .004), L5 R (≥0.074, AUC = 0.767, *P* = .004), sacral R (≥0.068, AUC = 0.793, *P* = .002), iliac R (≥0.076, AUC = 0.767, *P* = .004), and pubic R (≥0.049, AUC = 0.756, *P* = .006). The corresponding sensitivities were 82.4%, 70.6%, 82.4%, 64.7%, and 52.9%, with specificities of 69.6%, 78.3%, 69.6%, 91.3%, and 95.7%, respectively. The pretreatment lumbosacral *R*-value demonstrated particularly good predictive value.

**Table 4:** ROC curves analysis for grades ≥ 3 HT.

Parameter	cutoff	Apparent AUC	LOOCV AUC	LOOCV AUC (95% CI)	*P*-value	Sensitivity	Specificity
L4 R(BT)	≥0.089	0.769	0.724	[0.542–0.881]	.004	82.4%	69.6%
L5 R(BT)	≥0.074	0.767	0.737	[0.566–0.883]	.004	70.6%	78.3%
Sacral R(BT)	≥0.068	0.793	0.752	[0.590–0.891]	.002	82.4%	69.6%
Lliac R(BT)	≥0.076	0.767	0.711	[0.523–0.880]	.004	64.7%	91.3%
Pubic R(BT)	≥0.049	0.756	0.698	[0.519–0.864]	.006	52.9%	95.7%
L4 ΔR	≥0.050	0.737	0.696	[0.505–0.871]	.01	76.5%	78.3%
L5 ΔR	≥0.054	0.766	0.708	[0.525–0.868]	.004	58.8%	82.6%
Sacral ΔR	≥0.031	0.767	0.724	[0.560–0.877]	.004	94.1%	52.2%
Lliac R(AT)	≥0.039	0.774	0.729	[0.566–0.878]	.003	88.2%	56.5%
Sacral R(AT)	≥0.030	0.687	0.702	—	.04	82.4%	60.9%
L4 R(AT)	—	0.669	—	—	.07	—	—
L5 R(AT)	—	0.587	—	—	.35	—	—
Lliac ΔR	—	0.659	—	—	.09	—	—
Pubic R(AT)	—	0.492	—	—	.93	—	—
Pubic ΔR	—	0.651	—	—	.11	—	—
BM Grading (BT)	—	0.536	—	—	.70	—	—
BM Grading (AT)	—	0.430	—	—	.45	—	—

AT = after treatment, AUC = area under the curve, BT = before treatment, CI = confidence interval, cvAUC = cross-validated AUC from leave-one-out cross-validation, HT = hematologic toxicity.

To evaluate the stability and generalizability of these predictive models beyond the initial sample, LOOCV was performed. The cross-validated AUC (cvAUC) and its 95% CI for each significant predictor are reported in Table 4. For instance, the leading predictor, Sacral R(BT), maintained a cvAUC of 0.752 (95% CI: 0.590–0.891), indicating robust model performance.

## 4. Discussion

With advancements in radiotherapy techniques, the radiation dose to the BM of patients with gynecological malignancies has been reduced, effectively lowering the risk of HT.^[12]^ However, radiation-induced BM injury remains unavoidable, particularly in cases involving large volumes of low-dose pelvic irradiation.^[13]^ Consistent with prior reports estimating a 10% to 70% incidence of severe HT during CRT for advanced cervical cancer,^[14]^ our study found a significant increase in grade ≥ 2 HT post-CRT, with 17 patients (42.5%) experiencing acute grade 3 HT. Both qualitative and semi-quantitative *R* values of pelvic BM declined after CRT on imaging. This can likely be attributed to the fact that nearly half of the ABM resides in the pelvis, rendering it susceptible to radiation exposure. Such irradiation can damage the reticuloendothelial cell population within the BM, impairing its tracer uptake capacity and manifesting as the observed HT.^[15]^

While BM planar imaging allows direct visualization of whole-body BM activity distribution, its limited spatial and energy resolution may lead to inaccuracies due to inconsistent dosing, interference from liver/spleen activity, and subjective interpretation. Therefore, our study utilized SPECT/CT for BM imaging and employed the semi-quantitative index R for analysis. SPECT/CT provides a more intuitive 3-dimensional visualization of BM distribution across regions. Consistent with a previous investigation,^[16]^ ABM in the pelvic region was primarily distributed in the lumbar spine, sacrum, and iliac bones. Furthermore, patients undergoing CRT showed a significant decrease in semi-quantitative R values across the pelvic region, with a markedly greater reduction observed in the lumbosacral area (L4, L5, sacrum) compared to the Iliac and pubic bones. This conclusion is strengthened not only by conventional statistical significance but, more informatively, by a moderate effect size (partial η^2^ = 0.156), which underscores the magnitude of this inter-site disparity. Additionally, patients who developed BMS were characterized by significantly more attenuated decline in activity (Δ*R*) after CRT in the lumbosacral region compared to those without BMS, with large inter-group effect sizes (Cohen *d* predominantly ranging from −0.87 to −1.05). This suggests that CRT may exert a more pronounced impact on the lumbosacral BM in patients with advanced cervical cancer. Potential explanations include: first, the vertebrae are closer to the central radiation field during pelvic radiotherapy than the iliac and pubic bones; second, normal adult lumbar vertebrae and sacrum contain a higher proportion of red BM (approximately 25%), characterized by higher cellular turnover and metabolic activity. In contrast, the pelvis contains a greater proportion of fatty, yellow marrow. The significantly larger Δ*R* in the axial compartment likely reflects a more pronounced functional perturbation of this radiosensitive, hematopoietically active tissue following radiation exposure, providing an imaging-based, quantitative correlate to this fundamental physiological distinction; third, red BM is more concentrated in the lumbar and sacral vertebrae, whereas it is more dispersed in the flat iliac and pubic bones. Therefore, rather than focusing solely on sparing the ilium or iliac crest during radiotherapy, our findings suggest that prioritizing the preservation of lumbar and sacral vertebral marrow function during CRT, by minimizing radiation doses to the lumbosacral region as much as possible, could be a more effective strategy.

A primary objective of this exploratory study was to identify pelvic subregions where BM function correlates with acute HT. Similar to the findings of Meng et al., who reported no association between diagnosed age, FIGO stage, differentiation, and HT during CRT,^[17]^ our study also found no statistically significant differences in these clinical parameters between the BMS and NBMS groups. Previous studies have established a linear correlation between pelvic radiation dose/volume and short-term HT indicators.^[9,18–20]^ However, few studies have emphasized the relationship between specific pelvic subsites and HT. In 2006, Mell et al were the first to observe a stronger correlation between HT and the radiation dose to lumbosacral and lower pelvic BM compared to iliac BM in cervical cancer patients.^[16]^ Our analysis extended this by examining functional parameters. We found that both the qualitative grading of ^99m^Tc-SC BM imaging and semi-quantitative SPECT/CT parameters (*R* values) were negatively correlated with HT grades. Notably, the baseline *R* value at L4 showed the strongest correlation (*r* = −0.524). Furthermore, our stratified analysis revealed a distinct profile in patients who developed severe HT (BMS group): they exhibited significantly higher baseline BM activity (pretreatment R) in key lumbosacral sites (L4, L5, sacrum) and a significantly attenuated functional response post-CRT (greater Δ*R*) compared to the NBMS group. This counterintuitive association, wherein elevated pretreatment activity predicts higher toxicity risk, may be attributed to the intrinsic radiosensitivity of ABM. Regions demonstrating higher baseline ^99m^Tc-SC uptake likely correspond to areas with greater density and proliferative activity of hematopoietic cells. These actively cycling progenitor cells are known to be more susceptible to the cytotoxic effects of CRT.^[21]^ Consequently, despite an initially superior functional state, these more active marrow compartments may undergo a more profound functional suppression following CRT, thereby elevating the clinical likelihood of severe myelosuppression.

Interestingly, in this preliminary analysis, pretreatment lumbosacral R values demonstrated a better predictive potential for grade ≥ 3 HT than post-treatment values, Δ*R*, or baseline values from the iliac/pubis. Specifically, baseline L4 *R* ≥ 0.089, L5 *R* ≥ 0.074, and sacral *R* ≥ 0.068 emerged as potential high-risk thresholds. Higher baseline *R* values may indicate a greater concentration of ABM-derived macrophages, leading to higher ^99m^Tc-SC uptake and potentially greater radiosensitivity. The internal validation via leave-one-out cross-validation yielded stable cross-validated AUCs (e.g., 0.724 for L4, 0.737 for L5, 0.752 for Sacrum) close to the apparent AUCs, indicating robust model performance with minimal overfitting in our cohort. Therefore, there is a rationale to consider pretreatment pelvic BM SPECT/CT parameters, especially from the lumbosacral region, as potential imaging biomarkers for predicting acute HT. This could alert oncology teams to consider enhanced supportive measures for high-risk patients. However, these promising preliminary findings and the proposed predictive model require rigorous validation in larger, prospective, multi-center studies.

This study has several limitations that must be acknowledged, largely stemming from its exploratory, retrospective, single-center nature. First, the sample size (n = 40) is relatively small, which limits statistical power, increases the risk of Type II errors, and precludes meaningful subgroup analyses. The predictive thresholds and model performance metrics, although internally validated, require confirmation in larger, independent cohorts. Second, extended follow-up is necessary to observe potential long-term BM suppression and irreversible morphological changes induced by high-dose radiotherapy.^[16]^ Finally, the generalizability of our findings may be limited to patients with characteristics similar to our cohort and may not extend to those with severe anemia, poor performance status, or differing treatment protocols.

## 5. Conclusion

In conclusion, this study elucidates the spatially heterogeneous response of BM to CRT in cervical cancer, with the lumbosacral spine exhibiting preferentially greater functional suppression. Elevated baseline semi-quantitative parameters derived from ^99m^Tc-SC SPECT/CT, particularly those obtained from the lumbosacral region, demonstrate potential utility as stable, imaging-based predictors for the development of severe HT. It is acknowledged that other institutions have not yet implemented this imaging protocol; therefore, multi-center data are currently unavailable for incorporation. Nonetheless, our study has demonstrated that this imaging approach is feasible, simple, and cost-effective. These insights, gleaned through a robust statistical framework, suggest that routinely available functional imaging may inform personalized risk stratification in clinical practice. However, these promising yet preliminary findings necessitate confirmation and extension through rigorously designed, large-scale, prospective, multi-institutional investigations before broader application can be considered.

## Author contributions

**Conceptualization:** Chunmeng Chen, Jianqiang Wang, Qi Yang.

**Data curation:** Chunmeng Chen, ShanBing Wang, Jiapei Liu, Jie Liao.

**Formal analysis:** Chunmeng Chen, ShanBing Wang, Jiapei Liu, Jie Liao.

**Methodology:** Chunmeng Chen.

**Project administration:** Chunmeng Chen, ShanBing Wang.

**Writing – original draft:** Chunmeng Chen.

**Writing – review & editing:** Chunmeng Chen, Jianqiang Wang, Qi Yang.

**Resources:** ShanBing Wang, Jiapei Liu, Jie Liao.

## Supplementary Material
