# Supplementary material for: Bone marrow function in cervical cancer patients after concurrent chemoradiotherapy using 99mTc-SC SPECT/CT: A cross-sectional retrospective study
Source: Medicine (Baltimore). 2026 Feb 20;105(8):e47766. doi: 10.1097/MD.0000000000047766 (PMC12928878; doi:10.1097/MD.0000000000047766)
Supplement: Supplementary file 1 [file medi-105-e47766-s001.doc]

**Supplementary Table S1. Pairwise comparisons of ΔR values among five anatomical sites (with FDR correction)**

| **ComparisonPair** | **TestStatistic** | **Std.Error** | **Std.TestStatistic** | **P-value** | **Adj. P-value, FDRq-value** |
| --- | --- | --- | --- | --- | --- |
| △RC – △RQ | 13.000 | 12.941 | 1.005 | .315 | 1.000 |
| △RC – △Rs | 47.863 | 12.941 | 3.699 | < .001 | **< .001** |
| △RC – △R5 | 56.638 | 12.941 | 4.377 | < .001 | **< .001** |
| △RC – △R4 | 59.500 | 12.941 | 4.598 | < .001 | **< .001** |
| △RQ – △Rs | 34.863 | 12.941 | 2.694 | .007 | **.007** |
| △RQ – △R5 | 43.638 | 12.941 | 3.372 | .001 | **.001** |
| △RQ – △R4 | 46.500 | 12.941 | 3.593 | < .001 | **.001** |
| △Rs – △R5 | 8.775 | 12.941 | 0.678 | .498 | .498 |
| △Rs – △R4 | 11.638 | 12.941 | 0.899 | .369 | .369 |
| △R5 – △R4 | 2.863 | 12.941 | 0.221 | .825 | .825 |

***Note:* Post-hoc pairwise comparisons were performed using Dunn's test following significant one-way ANOVA (F(4, 195) = 9.010, P < 0.001, partial η² = 0.156) and Kruskal-Wallis test (χ²(4) = 35.14, P < 0.001). The Benjamini-Hochberg false discovery rate (FDR) procedure was applied to adjust P-values for multiple comparisons. An adjusted q-value < 0.05 was considered statistically significant (shown in bold).**
